# How can the response to volume expansion in patients with spontaneous respiratory movements be predicted?

**DOI:** 10.1186/cc4970

**Published:** 2006-07-17

**Authors:** Sarah Heenen, Daniel De Backer, Jean-Louis Vincent

**Affiliations:** 1 Department of Intensive Care, Erasme University Hospital, Free University of Brussels, Route de Lennik, 808, B-1070 Brussels, Belgium

## Abstract

**Introduction:**

The aim of the study was to evaluate the ability of different static and dynamic measurements of preload to predict fluid responsiveness in patients with spontaneous respiratory movements.

**Methods:**

The subjects were 21 critically ill patients with spontaneous breathing movements receiving mechanical ventilation with pressure support mode (*n *= 9) or breathing through a face mask (*n *= 12), and who required a fluid challenge. Complete hemodynamic measurements, including pulmonary artery occluded pressure (PAOP), right atrial pressure (RAP), pulse pressure variation (ΔPP) and inspiratory variation in RAP were obtained before and after fluid challenge. Fluid challenge consisted of boluses of either crystalloid or colloid until cardiac output reached a plateau. Receiver operating characteristics (ROC) curve analysis was used to evaluate the predictive value of the indices to the response to fluids, as defined by an increase in cardiac index of 15% or more.

**Results:**

Cardiac index increased from 3.0 (2.3 to 3.5) to 3.5 (3.0 to 3.9) l minute^-1 ^m^-2 ^(medians and 25th and 75th centiles), *p *< 0.05. At baseline, ΔPP varied between 0% and 49%. There were no significant differences in ΔPP, PAOP, RAP and inspiratory variation in RAP between fluid responders and non-responders. Fluid responsiveness was predicted better with static indices (ROC curve area ± SD: 0.73 ± 0.13 for PAOP, *p *< 0.05 vs ΔPP and 0.69 ± 0.12 for RAP, *p *= 0.054 compared with ΔPP) than with dynamic indices of preload (0.40 ± 0.13 for ΔPP and 0.53 ± 0.13 for inspiratory changes in RAP, *p *not significant compared with ΔPP).

**Conclusion:**

In patients with spontaneous respiratory movements, ΔPP and inspiratory changes in RAP failed to predict the response to volume expansion.

## Introduction

Fluid challenge is commonly performed in critically ill patients but the response is quite variable [1]. Inappropriate fluid administration can result in interstitial edema, which may have harmful consequences, especially in patients with respiratory failure. Measurements of cardiovascular pressures or volumes do not reliably predict fluid responsiveness [1], because a given value may be associated with preload dependence as well as preload independence.

Recently, dynamic evaluation of preload indexes has been introduced, on the basis of the observation that cyclic changes in intrathoracic pressure induced by mechanical ventilation can result in concurrent changes in stroke volume in preload-dependent, but not in preload-independent, patients. These dynamic indices of preload can better predict the individual response to fluid loading than static indices [1-5].

However, all these studies have been performed in patients receiving mechanical ventilation, well sedated and even paralyzed to avoid any spontaneous respiratory movements. But spontaneous respiratory movements, inspiratory as well as expiratory, can also influence venous flow, preload, and afterload [6,7]. Rooke and colleagues [8] reported in seven awake subjects that systolic pressure variation did not change in response to blood withdrawal or volume infusion, but cardiac output was not measured in these patients. Hence, cardiac output may have been maintained in these healthy subjects, as a result of an adrenergic reaction. In addition, the impact of respiratory movements in patients treated with mechanical ventilation may have more limited impact, as inspiration is still associated with an increase in pleural pressure.

We therefore designed this study to assess the value of several dynamic and static indices of preload as predictors of fluid responsiveness in spontaneously breathing patients, receiving mechanical ventilation in pressure support mode or breathing through a face mask.

## Materials and methods

### Ethical considerations

This study was approved by the local Ethical Committee, and informed consent was obtained from the patients or their relatives.

### Patients

This prospective study included 21 patients over a six month period. Inclusion criteria consisted of the need for fluid loading (for arterial hypotension, tachycardia, or oliguria) in a patient equipped with a central venous catheter and an arterial catheter, in whom cardiac output was determined by the thermodilution technique either with a pulmonary artery catheter (Vigilance; Edwards Lifesciences, Irvine, CA, USA) or by a modified arterial catheter (PiCCO; Pulsion, Munich, Germany). For inclusion, each patient had to show spontaneous breathing movements. Exclusion criteria were the following: age less than 18 years, pregnancy, and any significant cardiac arrhythmia. Fourteen patients were treated with vasoactive agents; no change in vasoactive treatment was allowed during the study period.

### Methods

Arterial pressure was measured with either radial or femoral arterial catheters. Cardiac output was measured with a pulmonary artery catheter (Swan–Ganz catheter, Vigilance 7.5 French; Edwards Lifesciences) in 20 patients and by transpulmonary thermodilution (PiCCO, PVPK, 5 French; Pulsion) in one patient. After calibration, all pressures were recorded on a computer system. We looked carefully at patient respiratory efforts and manually noted each breath initiation. Right atrial pressure was measured both at end-expiration (RAP_ee_) and end-inspiration (RAP_ei_). When available, pulmonary arterial pressures and pulmonary artery occluded pressure (PAOP) were also measured at end-expiration. The arterial pressure waveforms were measured on the computer system, and variation in pulse pressure (ΔPP) was calculated. The respiratory variation in RAP was calculated as RAP_ee _- RAP_ei_.

A complete set of hemodynamic measurements as well as blood sampling for arterial and mixed venous blood gases were obtained at baseline and before each volume expansion (VE). VE consisted of 500 ml of synthetic colloid (Voluven^®^, hydroxyethylstarch 6%; Fresenius, Bad Homburg, Germany) or 1,000 ml of crystalloid (Hartmann solution; Baxter, Lessines, Belgium) infused over 30 minutes. Hemodynamic measurements were obtained after each 250 ml aliquot. The VE was interrupted when the cardiac output did not increase further and PAOP or RAP increased by more than 3 mmHg. At the end of VE, another complete set of hemodynamic measurements, including venous and arterial blood gases, was obtained. A rise of 15% or more in cardiac output between baseline and final measurements defined the responders.

### Statistics

As data were not normally distributed, non-parametric statistical tests were used; data are presented as medians, with 25th and 75th centiles in parentheses. The effects of VE on the hemodynamic variables were analyzed with the Wilcoxon rank test. Baseline values for responders and non-responders were compared by using the Mann–Whitney test. Spearman's correlations were used to analyze the relationship between baseline measurements and changes in cardiac index (ΔCI). Receiver operating characteristics (ROC) curves were used to evaluate the predictive value of the various indices on fluid responsiveness. ROC curve area are presented as area ± SD. A *p *value less than 0.05 was considered significant.

## Results

Patient characteristics are shown in Table 1. Nine patients were ventilated in pressure support mode and 12 were breathing spontaneously by means of a face mask. Four of the 21 patients received colloids (500 (437 to 500) ml) and 17 received crystalloids (1,000 (750 to 1,000) ml). Fluid challenge was stopped either because cardiac output failed to increase initially (*n *= 2) or because it reached a plateau (*n *= 19), in the presence of an increase in RAP or PAOP by at least 3 mmHg. Fluid infusion was stopped after one aliquot in two patients, two aliquots in six, three aliquots in three, and four aliquots in ten. None of the patients required more than four aliquots. Seventeen patients survived. The effects of VE are shown in Table 2. Cardiac index (CI) increased from 3.0 (2.3 to 3.5) to 3.5 (3.0 to 3.9) l minute^-1 ^m^-2 ^(*p *< 0.05), but it increased by more than 15% in only nine patients.

At baseline, ΔPP varied between 0 and 49%. There were no significant differences in ΔPP, PAOP at end-expiration, RAP_ee _and inspiratory variation in RAP between responders and non-responders (Table 3).

**Figure 1:**
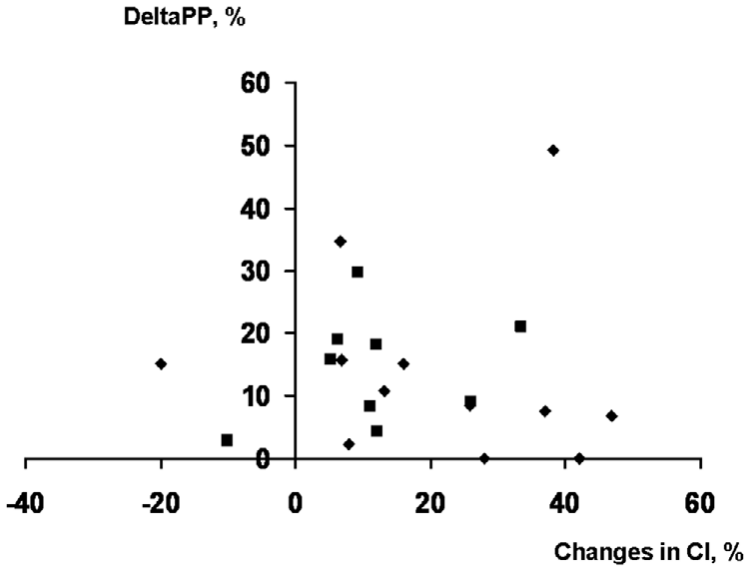
Relation between the ΔPP and the maximal ΔCI after volume expansion. This relationship was not significant (*R*^2 ^= 0.02, *p *= 0.94). Diamonds, patients breathing through a face mask; squares, patients receiving pressure support ventilation. ΔCI, change in cardiac index; ΔPP, pulse pressure variation.

**Figure 2:**
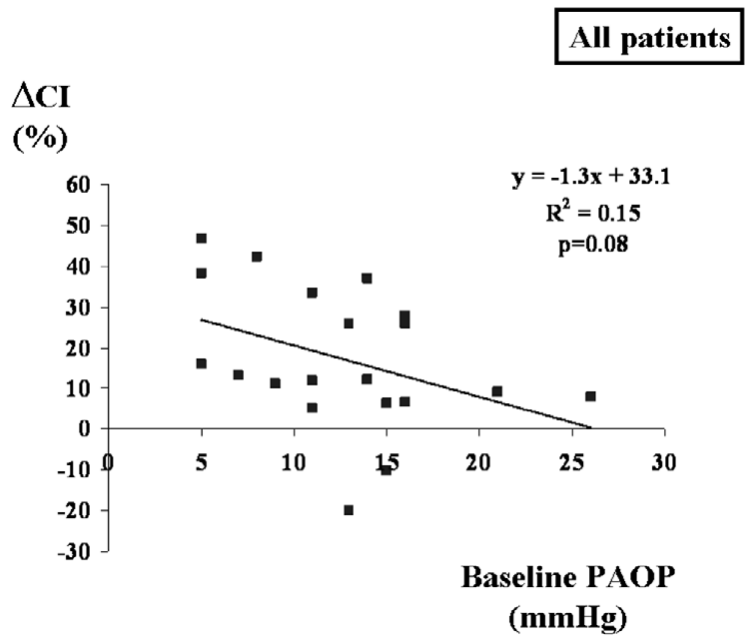
Relation between PAOP at baseline and maximal ΔCI during volume expansion. ΔCI, change in cardiac index; PAOP, pulmonary artery occluded pressure.

**Figure 3:**
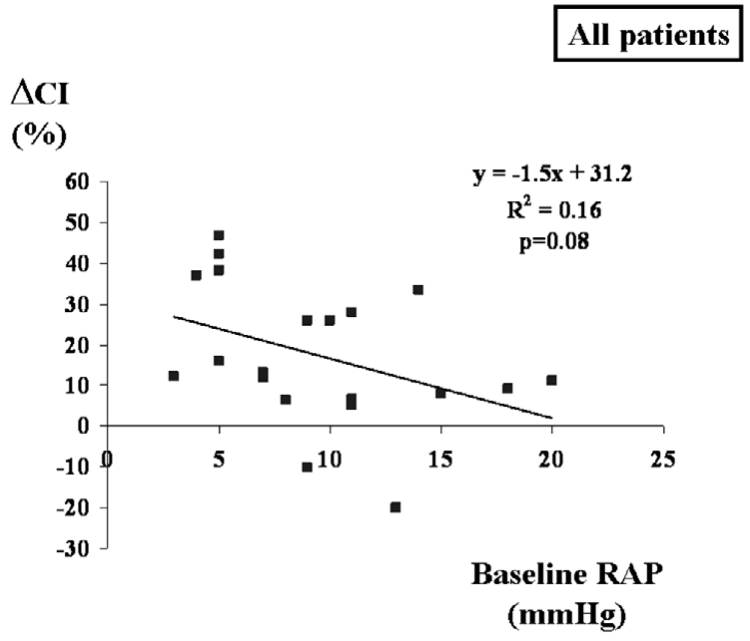
Relation between RAP at baseline and maximal ΔCI during volume expansion. ΔCI, change in cardiac index; RAP, right atrial pressure.

**Figure 4:**
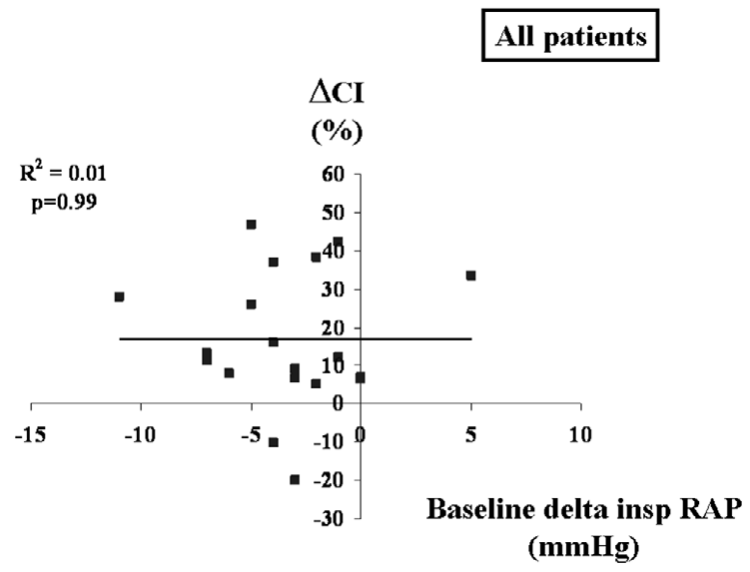
Relation between ΔRAP at baseline and maximal ΔCI during volume expansion. ΔCI, change in cardiac index; ΔRAP, respiratory variation in right atrial pressure.

No significant relationship was found between the change in CI (ΔCI) during VE and ΔPP baseline for the entire group or for the subgroups (mechanical ventilation and spontaneous breathing; Figure 1). The relationships between PAOP at baseline and ΔCI (Figure 2) and RAP_ee _at baseline and ΔCI (Figure 3) were not significant (*p *= 0.08 for each). There was no relationship between the inspiratory variation in RAP at baseline and ΔCI (Figure 4).

**Figure 5:**
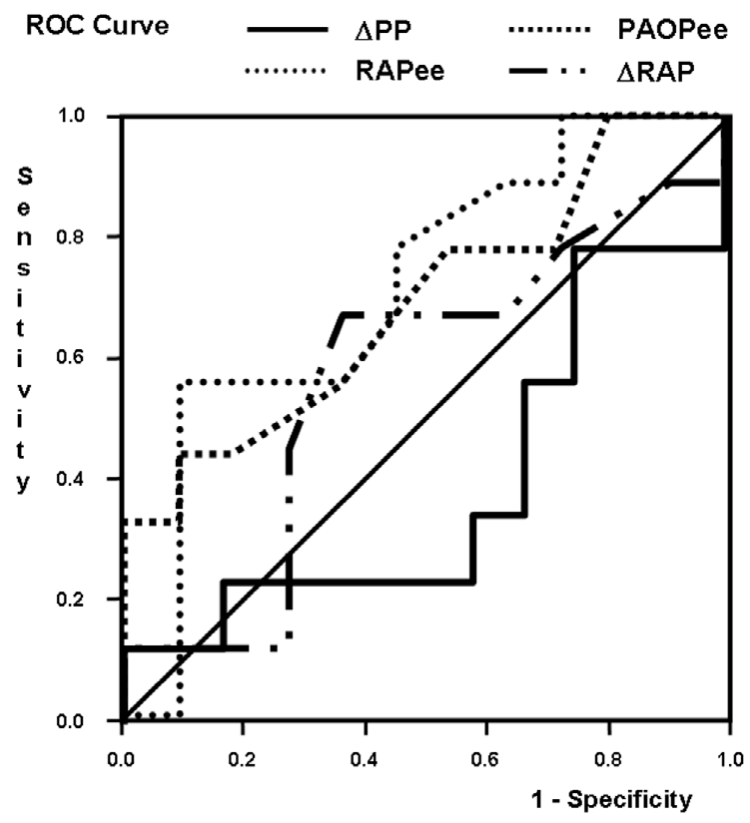
Prediction of fluid responsiveness by ΔPP, PAOP_ee_, RAP_ee _and ΔRAP. The receiver operating characteristics (ROC) curve area was significantly larger for pulmonary artery occluded pressure at end-expiration (PAOP_ee_) than for pulse pressure variation (ΔPP; *p *< 0.05). ΔRAP, inspiratory variation in RAP; RAP_ee_, right atrial pressure at end-expiration.

**Figure 6:**
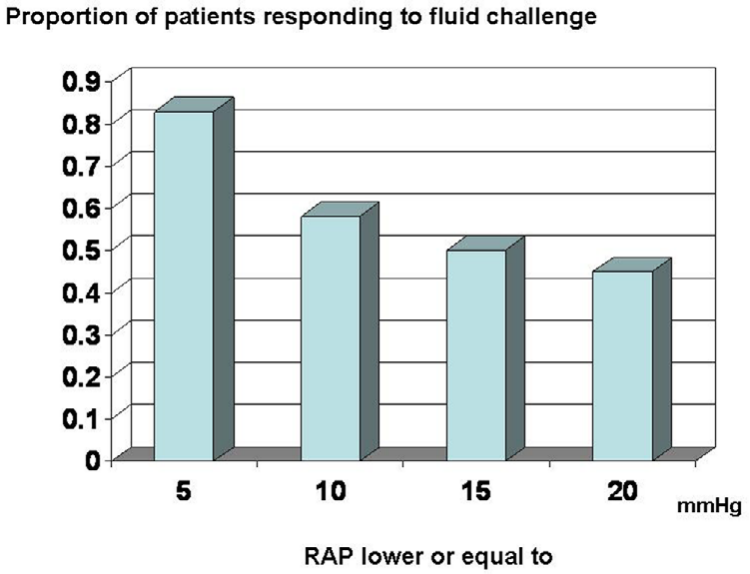
Relationship between right atrial pressure (RAP) and the likelihood of responding to fluid challenge.

The predictive value of the various indices on fluid responsiveness was compared (Figure 5). The ROC curve area was larger for static indices (0.73 ± 0.13 for PAOP, *p *< 0.05 compared with ΔPP; and 0.69 ± 0.12 for RAP_ee_, *p *= 0.054 compared with ΔPP) than for dynamic indices of preload (0.40 ± 0.13 for ΔPP, and 0.53 ± 0.13 for inspiratory variation in RAP; *p *not significant compared with ΔPP). The likelihood of a response to fluids was highest at low values of RAP (Figure 6) and PAOP (data not shown), and decreased progressively when RAP and PAOP were higher.

Several subgroup analyses were conducted to provide a better definition of the potential factors influencing ΔPP and inspiratory variation in RAP. No patient had evidence of right ventricular dysfunction, either before or after fluid challenge. We noticed active expiratory efforts in four patients, but excluding these patients did not alter the results (data not shown). The ROC curve area of ΔPP was 0.64 ± 0.26 in patients receiving mechanical ventilation, and 0.29 ± 0.17 in patients breathing through a face mask (*p *= 0.25). For inspiratory variation in RAP, only three patients had no decrease in CVP during inspiration, and one of these responded to fluid challenge (negative predictive value of 66%). In comparison, 8 of the 18 patients with a 1 mmHg inspiratory decrease in RAP responded to fluids (the positive predictive value was 44%). Four patients presented respiratory efforts insufficient to generate an inspiratory decrease in PAOP of more than 2 mmHg, and excluding these patients from the analysis did not alter our results (data not shown).

## Discussion

Important questions are the following. Which indices can be used to predict fluid responsiveness in patients with respiratory movements [9]? In particular, can dynamic indexes of preload be useful in this context? The data reported so far have been obtained for patients who were deeply sedated and even paralyzed [2,10], a situation that physicians prefer to avoid whenever possible [11]. Our results show that ΔPP cannot predict fluid responsiveness reliably in patients who either trigger the respirator or breathe spontaneously. Furthermore, its predictive value is inferior to that of static measurements of cardiac filling pressures.

It has indeed been proposed that ΔPP (and other indices of ventilation-induced stroke volume variations) may not apply in patients breathing spontaneously [6,7], but this has never been shown. Pinsky and colleagues [12] reported that spontaneous respiratory efforts in dogs increased transmural right atrial pressure and right ventricular stroke volume, whereas positive pressure ventilation induced inverse changes, thus suggesting that breathing movements and positive pressure ventilation may both be used to evaluate heart-lung interactions and to predict fluid responsiveness. However, our study indicates that the capacity of ΔPP to predict changes in cardiac index during fluid challenge is inaccurate in the presence of spontaneous respiratory movements.

Spontaneous respiratory movements can affect ΔPP through different pathways. First, respiratory changes in alveolar and pleural pressure are lower during spontaneous breaths than during mechanically assisted breaths. However, this factor may only account for patients breathing spontaneously through a face mask. Patients ventilated with pressure support ventilation experienced a range of driving pressures similar to those observed in other studies [13]. Second, active expiratory movements, which can occur both during spontaneous breathing and during mechanical ventilation, can alter the cyclic changes in alveolar pressure. The active expiratory contraction of abdominal muscles flushes blood from the abdominal compartment into the thorax, increasing the right ventricular preload and later the LV preload. Active expiration also induces a decrease in left ventricular afterload. This may counterbalance the cyclic modifications induced by the passive changes in intrathoracic pressure occurring in mechanically ventilated patients without spontaneous breathing movements. These changes may result in both false negative and false positive tests. Third, the respiratory rate may be higher in patients with spontaneous respiratory movements, so that the number of cardiac beats per respiratory cycle may be reduced, and hence the chance to detect respiratory variations in stroke volume. Finally, patients under less sedation may also experience variations in cardiac output independently of their preload status. They may be more sensitive to various stimuli (such as pain, noise, anxiety, or dyspnea), resulting in transient increases in oxygen consumption and consequently in cardiac output [14]. This could have happened at any time during the evaluation of the response to VE, affecting its interpretation.

In contrast to our expectations, respiratory variations in RAP were also not predictive of the response to fluid loading. Several factors may explain this finding. First, Magder and colleagues [15,16] demonstrated the usefulness of this index in patients who had no respiratory support at all. Indeed, Magder and colleagues evaluated the respiratory changes in RAP either in patients breathing spontaneously or after a brief disconnection from the ventilator in patients who were under pressure support ventilation. In our study, some patients were receiving pressure support and we decided not to disconnect these patients from the ventilator to avoid de-recruitment, and also because measurements obtained off ventilatory support may not reflect the situation during respiratory support. Magder and colleagues [17] showed, at an individual level, that respiratory changes in RAP were not predictive of changes in cardiac index after application of positive end-expiratory pressure. Second, we did not exclude any patient from this analysis, whereas Magder and colleagues [15,16] used this index only when patients were able to generate inspiratory efforts sufficient to decrease PAOP by 2 mmHg. In our study, only four patients had respiratory efforts insufficient to generate a 2 mmHg decrease in PAOP; removing these patients from the analysis did not improve the performance of the test. Finally, small errors in measurements can interfere with this index. Indeed, a positive test is defined as an inspiratory decrease in RAP by 1 mmHg. This level is far below the precision of measurements of CVP in patients with respiratory movements, as shown by Hoyt and colleagues [18].

Interestingly, the static variables were slightly better predictors of the response to a VE. This is also reflected by the inverse relationship between filling pressures and changes in CI, but this relationship was quite loose. As expected, no cut off value could be found and the differences in these indices between responders and non-responders were not significant.

We did not evaluate volumetric indices of preload, and these indices may perform better than pressure measurements, especially in the presence of diastolic dysfunction. These should be evaluated in further studies.

In the absence of a clear cut off value allowing reliable prediction of fluid responsiveness in the individual patient, the physician is left with the option of performing a fluid challenge; that is, testing the system. A possible alternative would be to perform a passive leg-raising test but this would require using a fast response methodology for cardiac output measurements. Of course, fluid challenge may fail to increase cardiac output, but the risks of performing a fluid challenge are basically the same whether or not cardiac index increases, because ventricular volumes and pressures increase in response to fluid (unless there is instantaneous elimination by the kidneys; however, in that case, preload is not affected). One might expect cardiac filling pressures to increase more in non-responders, but it would be limited as an increase in RAP or PAOP (or a volumetric index) is used as a safety limit. So the basic difference between a successful and a failed fluid challenge is that in responders the benefit is supposed to outweigh the risks, but the risks are similar. These risks are probably limited when fluid challenge is performed cautiously, using repeated aliquots of fluids over a short period and re-evaluating the hemodynamic situation before administration of the next bolus. Matejovic and colleagues [19] reported that extravascular lung water did not increase during a carefully conducted fluid challenge aimed at optimizing cardiac output and using intravascular pressures as safety rules in patients with sepsis and acute lung injury.

The fluid challenge technique we used is standardized, but it included various amounts of either colloid or crystalloid as recommended clinically. Even though colloids and crystalloids may have different volume expansion properties, fluid challenge was adapted to the hemodynamic response [20]. Thus, in each case, we attempted to achieve a maximal stroke volume, regardless of the amount of fluid required to achieve this goal. This method takes into account the fact that each patient has his or her own Starling relationship, a well established finding in critically ill patients [21], explaining why a definite amount of fluid does not achieve the same hemodynamic effect in each patient. This is also the rationale for using dynamic indices of preload; otherwise static measurements of preload would have good predictive value of fluid responsiveness. This method also ensures that the absence of an increase in CI is not due to insufficient fluid loading.

## Conclusion

In patients with spontaneous respiratory movements, ΔPP and inspiratory variations in RAP failed to predict the response to volume expansion.

## Key messages

• 	Pulse pressure variation and inspiratory variation in RAP failed to predict the response to fluid challenge in patients with spontaneous respiratory movements.

• 	Filling pressures are a slightly better predictor of fluid responsiveness than dynamic indices of preload, but no cutoff value was detected.

• 	Fluid challenge should be performed carefully when clinically indicated.

## Abbreviations

CI = cardiac index; ΔCI = change in cardiac index; ΔPP = pulse pressure variation; PAOP = pulmonary artery occluded pressure; RAP = right atrial pressure; RAP_ee _= RAP at end-expiration; RAP_ei _= RAP at end-inspiration; ROC = receiver operating characteristics; VE = volume expansion.

## Competing interests

SH declares no conflict of interest related to the current work; DDB and JLV have received research grants and/or material from Edwards Healthcare, LiDCO, and Pulsion.

## Authors' contributions

SH collected the data and contributed to the analysis of the data and writing of the manuscript. DDB designed the study collected the data and contributed to the analysis of the data and writing of the manuscript. JLV contributed to the analysis of the data and writing of the manuscript. All authors read and approved the final manuscript.

## Figures and Tables

**Table 1:** Characteristics of general population

Parameter	Value
Age (years)	74 (61–78)
Weight	80 (65–90)
Survivors, *n *(%)	17 (80)
Surgical, *n *(%)	15 (71)
Heart surgery	11
AAA	4
Medical	6
Sepsis	4
Intestinal bleeding	2
Vasoactive drugs	14
Dobutamine, *n*; dose, μg kg^-1 ^minute^-1^	8; 5 (3–8)
Dopamine	6; 15 (14–20)
Norepinephrine	4; 0.4 (0.3–0.6)
Sodium nitroprusside	2; 20–120
Mechanical ventilation, *n *(%)	9 (43)
Respiratory rate, minute^-1^	24 (19–27)
Cardiac beat per breath, *n*	4.2 (3.5–5.4)
PEEP (*n *= 9), cmH_2_O	5 (5–5)
Pressure support level (*n *= 9), cmH_2_O	22 (12–25)
FiO_2_	0.5 (0.4–0.5)

Ranges in parentheses are 25th to 75th centiles. AAA, abdominal aortic aneurysm; FiO_2_, fraction of inspired oxygen; PEEP, positive end-expiratory pressure.

**Table 2:** Evolution of the hemodynamic variables after VE

Parameter	Value	
	Baseline	After VE
Temperature, °C	37.1 (36.7–37.9)	37.0 (36.7–37.7)
Cardiac frequency, minute^-1^	94 (83–105)	97 (82–111)
Mean arterial pressure, mmHg	73 (67–81)	75 (69–83)
ΔPP, %	11 (7–18)	9 (4–16)
Mean PAP, mmHg	20 (14–25)	22 (18–29)
PAOP_ee_, mmHg	13 (9–15)	15 (10–19)^a^
RAP_ee_, mmHg	9 (5–12)	10 (7–13)^b^
Inspiratory ΔRAP, mmHg	-3 (-5 to -1)	-2 (-4 to 0)
Cardiac index, l minute^-1 ^m^-2^	3.0 (2.3–3.5)	3.5 (3.1–3.9)^a^
Oxygen delivery, ml minute^-1 ^m^-2^	402 (353–432)	434 (379–518)^b^
Oxygen consumption, ml minute^-1 ^m^-2^	138 (126–153)	145 (92–156)
Arterial pH	7.42 (7.38–7.45)	7.42 (7.39–7.48)
PaCO_2_	38 (34–39)	38 (37–39)
PaO_2_	99 (86–117)	96 (85–114)
SaO_2_	99 (98–99)	99 (97–100)
SvO_2_	67 (60–72)	71 (63–74)
Hemoglobin, g/dl	9.1 (8.9–12.0)	9.0 (8.1–11.2)^b^
Lactate, mEq/l	1.4 (1.2–1.9)	1.2 (1.0–1.5)^b^

Ranges in parentheses are 25th to 75th centiles. ΔPP, pulse pressure variation; ΔRAP, variation in right atrial pressure; PAOP_ee_, pulmonary arterial occluded pressure at end-expiration; PAP, pulmonary arterial pressure; RAP_ee_, right atrial pressure at end-expiration; PaCO_2_, arterial partial pressure of CO_2_; PaO_2_, arterial partial pressure of oxygen; SaO_2_, arterial oxygen saturation; SvO_2_, mixed venous oxygen saturation; VE, volume expansion. ^a^*p *< 0.01, and ^b^*p *< 0.05 compared with baseline.

**Table 3:** Baseline values in responders versus non-responders

Parameter	Value	
	Non-responders (*n *= 12)	Responders (*n *= 9)
Temperature, °C	36.9 (36.6–37.9)	37.2 (36.9–38.0)
Heart rate, minute^-1^	96 (79–105)	93 (88–110)
Mean arterial pressure, mmHg	74 (67–81)	70 (68–79)
ΔPP, %	15 (6–19)	9 (5–16)
Mean PAP, mmHg	21 (18–25)	17 (15–26)
PAOP_ee_, mmHg	14 (11–16)	11 (5–15)
RAP_ee_, mmHg	11 (7–15)	5 (5–10)
Inspiratory ΔRAP, mmHg	-3 (-5 to -1)	-4 (-5 to -2)
Cardiac index, l minute^-1 ^m^-2^	3.2 (2.7–3.5)	2.7 (2.3–3.4)
Arterial pH	7.39 (7.37–7.45)	7.44 (7.42–7.47)^a^
PaCO_2_	38 (36–40)	36 (31–39)
PaO_2_	97 (85–113)	108 (89–125)
SaO_2_	99 (97–99)	99 (98–99)
SvO_2_	67 (57–72)	67 (64–74)
Hemoglobin, g/dl	9.0 (8.8–11.9)	10.4 (8.7–12.5)
Lactate, mEq/l	1.6 (1.4–2.4)	1.3 (1.1–1.4)

ΔPP, pulse pressure variation; ΔRAP, variation in right atrial pressure; PaCO_2_, arterial partial pressure of CO_2_; PaO_2_, arterial partial pressure of oxygen; PAOP_ee_, pulmonary arterial occluded pressure at end-expiration; PAP, pulmonary arterial pressure; RAP_ee_, right atrial pressure at end-expiration; SaO_2_, arterial oxygen saturation; SvO_2_, mixed venous oxygen saturation. Ranges in parentheses are 25th to 75th centiles. ^a^*p *< 0.05 between the two groups.
